# A Genotyping/Phenotyping Approach with Careful Clinical Monitoring to Manage the Fluoropyrimidines-Based Therapy: Clinical Cases and Systematic Review of the Literature

**DOI:** 10.3390/jpm10030113

**Published:** 2020-09-03

**Authors:** Valeria Conti, Emanuela De Bellis, Valentina Manzo, Francesco Sabbatino, Francesco Iannello, Fabrizio Dal Piaz, Viviana Izzo, Bruno Charlier, Berenice Stefanelli, Martina Torsiello, Teresa Iannaccone, Albino Coglianese, Francesca Colucci, Stefano Pepe, Amelia Filippelli

**Affiliations:** 1 Department of Medicine, Surgery and Dentistry “Scuola Medica Salernitana”, University of Salerno, 84081 Baronissi, Italy; vconti@unisa.it (V.C.); fsabbatino@unisa.it (F.S.); fdalpiaz@unisa.it (F.D.P.); vizzo@unisa.it (V.I.); bcharlier@unisa.it (B.C.); tiannaccone@unisa.it (T.I.); albino.cog@gmail.com (A.C.); spepe@unisa.it (S.P.); afilippelli@unisa.it (A.F.); 2 Clinical Pharmacology and Pharmacogenetics Unit, University Hospital “San Giovanni di Dio e Ruggi d’Aragona”, 84131 Salerno, Italy; 3 Postgraduate School in Clinical Pharmacology and Toxicology, University of Salerno, 84081 Baronissi, Italy; e.debellis93@gmail.com (E.D.B.); b.stefanelli@studenti.unisa.it (B.S.); m.torsiello5@studenti.unisa.it (M.T.); francescacolucci90@gmail.com (F.C.); 4 Oncology Unit, University Hospital “San Giovanni di Dio e Ruggi d’Aragona”, 84131 Salerno, Italy; 5 Postgraduate School in Clinical Pharmacology and Toxicology, University of Campania “L. Vanvitelli”, 80138 Naples, Italy; francesco.iannello@hotmail.it

**Keywords:** DPD, DPYD polymorphisms, pharmacogenetics, fluoropyrimidines, personalised medicine, therapeutic drug monitoring

## Abstract

Fluoropyrimidines (FP) are mainly metabolised by dihydropyrimidine dehydrogenase (DPD), encoded by the *DPYD* gene. FP pharmacogenetics, including four *DPYD* polymorphisms (*DPYD*-PGx), is recommended to tailor the FP-based chemotherapy. These polymorphisms increase the risk of severe toxicity; thus, the *DPYD*-PGx should be performed prior to starting FP. Other factors influence FP safety, therefore phenotyping methods, such as the measurement of 5-fluorouracil (5-FU) clearance and DPD activity, could complement the *DPYD*-PGx. We describe a case series of patients in whom we performed *DPYD*-PGx (by real-time PCR), 5-FU clearance and a dihydrouracil/uracil ratio (as the phenotyping analysis) and a continuous clinical monitoring. Patients who had already experienced severe toxicity were then identified as carriers of *DPYD* variants. The plasmatic dihydrouracil/uracil ratio (by high-performance liquid chromatography (HPLC)) ranged between 1.77 and 7.38. 5-FU clearance (by ultra-HPLC with tandem mass spectrometry) was measured in 3/11 patients. In one of them, it reduced after the 5-FU dosage was halved; in the other case, it remained high despite a drastic dosage reduction. Moreover, we performed a systematic review on genotyping/phenotyping combinations used as predictive factors of FP safety. Measuring the plasmatic 5-FU clearance and/or dihydrouracil/uracil (UH2/U) ratio could improve the predictive potential of *DPYD*-PGx. The upfront DPYD-PGx combined with clinical monitoring and feasible phenotyping method is essential to optimising FP-based chemotherapy.

## 1. Introduction

Fluoropyrimidines (FP), including 5-fluorouracil (5-FU) and its oral prodrug capecitabine, are cytotoxic antineoplastic agents belonging to the class of antimetabolites. They are commonly used to treat solid cancer types such as gastrointestinal, head-neck and breast cancers associated or not to other chemotherapeutics and both cytotoxic and biologic drugs [1,2]. The administration of the FP may cause severe, even life-threatening, adverse drug reactions (ADR), including myelosuppression, mucositis/stomatitis, diarrhoea and hand–foot syndrome (HFS). Indeed, it has been estimated that an increased risk of severe ADR (grade > 2) involves 10–30% of treated patients, although these data greatly depend on the therapeutic regimen used [1,3,4].

The rate-limiting step of FP catabolism is the conversion of fluorouracil to dihydrofluorouracil, which is catalysed by an enzyme called dihydropyrimidine dehydrogenase (DPD), encoded by a highly polymorphic gene (i.e., *DPYD*). Several single-nucleotide polymorphisms (SNPs) have been associated to an alteration of the *DPYD* sequence, and some of them may determine a partial or complete DPD deficiency, leading to FP severe toxicity [1].

The Clinical Pharmacogenetics Implementation Consortium (CPIC) and the Dutch Pharmacogenetics Working Group (DPWG) [5,6] have published guidelines for FP dosing based on the pharmacogenetic testing of four *DPYD* polymorphisms that are DPYD*2A (rs3918290), DPYD*13 (rs55886062), *DPYD c.2846A>T* (rs67376798) and *c.1129-5923C>G* (rs75017182 and *HapB3*). The latter is the most common variant, with ~4% allelic frequency; the DPYD*2A, c.2846A>T and DPYD*13 are present in ~2.0%, 1.4% and 0.1%, respectively, of Caucasian patients [5]. Recently, a new polymorphism, DPYD*6 (rs1801160), has been associated with both gastrointestinal and haematological FP-ADR [7].

Notably, other *DPYD* genetic variants may lead to dangerous clinical consequences, although their frequency is very low [5,6,8].

With the main aim of reducing the risk of severe FP-induced toxicity, the CPIC and DPWG have implemented a gene activity score (*DPYD*-AS), which ranges from 0 (complete DPD deficiency) to 2 (normal DPD activity). In patients who are homozygous for one or more of the aforementioned SNPs, the recommendation is to avoid the use of FP. However, if alternative drugs are not considered a suitable option, the FP dosage should be markedly reduced while establishing a therapeutic drug-monitoring (TDM) approach. Patients who are heterozygous should receive a 50% dose reduction at the first cycle of chemotherapy, followed by a titration dose, while monitoring the patient’s clinical conditions and possibly performing TDM [5,6].

However, it has been estimated that 30–50% of the patients experience severe ADR, despite not having a DPD deficit associated with such *DPYD* polymorphisms. In fact, there are several factors, including comorbidities, polytherapy, other variants in the *DPYD* and other genes, that can play an important role [9]. Besides *DPYD* polymorphisms, two SNPs in the 5,10-methylenetetrahydrofolate reductase (*MTHFR*) gene [10] and a tandem repeat in the thymidylate synthase enhancer region (*TYMS-TSER*) could concur in predicting FP-related toxicity [11]. Moreover, a SNP in glutathione S-transferase-p1 (*GSTP1*) has been suggested as a genetic factor able to influence the response to oxaliplatin, a drug frequently administered with FP [12].

Several strategies complementing the *DPYD* pharmacogenetics (*DPYD*-PGx) have been proposed to prevent FP-related severe ADR associated with DPD deficit. Among others, the measurement of the plasmatic dihydrouracil/uracil ratio (UH_2_/U) and the monitoring of 5-FU clearance are considered valid approaches [13,14]. Here, we reported clinical cases in whom a combined genotyping/phenotyping approach, together with careful clinical monitoring was used to optimise the FP-based treatment. In addition, we performed a systematic review of the literature concerning the use of *DPYD*-PGx, together with phenotyping methods to personalise such chemotherapy.

## 2. Materials and Methods

### 2.1. Case Series

We describe the cases of eleven oncological patients of the Campania Region (Italy) treated with FP-based chemotherapy. They were enrolled in an ongoing study (ethics committee approval n. 4_r.p.s.o.) whose main aim was to investigate the association between *DPYD* SNPs and other genetic variants with FP-related severe toxicity. To manage the therapy of these eleven patients, we used a combined genotyping/phenotyping approach with clinical monitoring.

A careful clinical monitoring was carried out by interviewing patients and checking the results of their blood counts at each cycle of chemotherapy. The ADR were recorded and graded according to the Common Terminology Criteria for Adverse Events (CTC-AE) version 5.0 [15]. The Response evaluation criteria in solid tumors (RECIST) were used to assess the treatment response [16].

All enrolled patients were genotyped for the recommended *DPYD* SNPs (DPYD*2A, DPYD*13, *DPYD* 2846A>T and c.1129-5923C>G) as part of the clinical practice. Besides this pharmacogenetic testing (*DPYD*-PGx) performed by real-time PCR with allelic discrimination, the SNPs MTHFR-C677T and -A1298C were analysed using pyrosequencing technology and the TYMS-TSER variant by classical PCR and agarose gel-based electrophoresis.

A phenotyping analysis was carried out by determining the plasmatic UH2/U ratio, as an indirect measurement of DPD activity, using high-performance liquid chromatography (HPLC) [17]. Moreover, in three patients, a 5-FU pharmacokinetic analysis was also performed to determine the plasmatic 5-FU clearance by using ultra-high-performance liquid chromatography combined with tandem mass spectrometry (UHPLC-MS/MS) [18]. Written informed consent was obtained from the patients for participation in the study, as well as publication of their data.

### 2.2. Systematic Review

#### 2.2.1. Search Strategy

A systematic review was performed using the PubMed, Scopus and Cochrane databases from inception to 3 July 2020. The following keywords and Medical Subject Headings (MeSH) terms were used for the search: *DPYD* polymorphism, dihydropyrimidine dehydrogenase deficiency, 5-FU clearance and dihydrouracil/uracil ratio.

**Figure 1:**
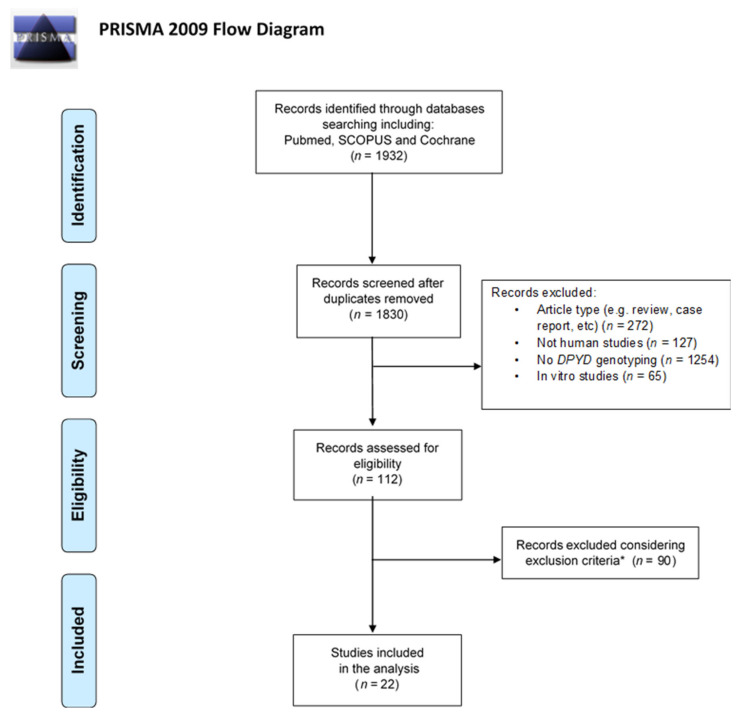
Flowchart of the databases searched. * Exclusion criteria: (1) studies not including at least one of the four recommended *DPYD* polymorphisms and (2) other study designs (e.g., allelic frequencies estimation, no patients and methods for genotyping).

The Preferred Reporting Items for Systematic Reviews and Meta-analyses (PRISMA) guidelines were applied [19]. A total of 1932 studies were retrieved from the searched databases. After the removal of duplicates and then article types such as reviews, metanalyses, case reports, etc., 112 articles were screened. Afterward, considering the inclusion and exclusion criteria, 22 articles were included in the analysis. The flowchart of the literature screening is reported in Figure 1.

#### 2.2.2. Eligibility Criteria

The search was limited to studies conducted in human beings and published in the English language. Both observational and randomised clinical studies were eligible. Only the studies enrolling patients treated with FP and showing results of *DPYD*-PGx, analysing at least one of the four SNPs recommended in the CPIC and DPWG guidelines, have been included in the analysis.

#### 2.2.3. Article Selection

First, articles were selected by reading their titles and abstracts. Then, the full text of all articles considered eligible was read. The authors (Valeria Conti and Berenice Stefanelli) carried out this work independently and discussed when there was a disagreement about the relevance of an article.

#### 2.2.4. Data Extraction

Clinical, genetic and biochemical data were extracted from the articles. The factors considered were: study design, sample size, *DPYD* genotyping combined with phenotyping methods and/or clinical monitoring.

## 3. Results

### 3.1. Cases Presentation

Case 1 was a Caucasian 55-year-old male former smoker with a history of hypertension. The patient had stage IV colorectal adenocarcinoma with metastases in the lymph nodes, lungs, liver and kidneys. The tumour mutational profile identified no mutations in the *KRAS*, *NRAS* or *BRAF* genes. The patient was treated with the combination of 5-FU, leucovorin and oxaliplatin (FOLFOX6) regimen, plus cetuximab. After three cycles of chemotherapy, the patient reported grade 1 thrombocytopenia and paraesthesia; grade 2 stomatitis, rash and leukopenia and grade 3 neutropenia and mucositis.

A post-therapeutic *DPYD*-PGx was performed, revealing that the patient was heterozygous for DPYD*2A. Moreover, the patient was homozygous (TT) for *MTHFR-C677T*, homozygous *TYMS-TSER-2R/2R* and homozygous (AA) for *GSTP1-A313G*. The plasmatic UH2/U ratio was 4.52. Based on these results and the reported toxicity, both the 5-FU and cetuximab doses were reduced. Specifically, the total dosage of 5-FU was reduced to 50%, according to the CPIC and DPWG guidelines.

At the fourth cycle of therapy, the pharmacokinetic analysis revealed a trough 5-FU plasma concentration of 950 ng/mL. The CT scan demonstrated an overall stable disease, according to the RECIST criteria v1.1. The patient was still treated with the same doses of 5-FU. At the sixth cycle of therapy, the 5-FU plasma concentration was 400 ng/mL. The following cycles (fifth to eighth) of chemotherapy were administered at the same drug doses. A new CT scan demonstrated no evidence of disease progression. The ADR were grade 1 leukopenia, neutropenia, thrombocytopenia and mucositis and grade 2 HFS. Following a further two cycles of therapy, the reported ADR were grade 1 paraesthesia, erythematous maculopapular rash and grade 2 cutaneous and mucous fissures. Lastly, following a further two treatment cycles, a new CT scan showed disease progression. The treatment was stopped, and the administration of a new chemotherapeutic regimen was planned. Sadly, the patient died before starting a second line of treatment.

Case 2 was a Caucasian 48-year-old male with no comorbidity. He had stage IV colorectal adenocarcinoma with metastases in the lymph nodes and liver. The tumour mutational profile highlighted the presence of a *KRAS* mutation; thus, a treatment with the FOLFOX6 regimen plus bevacizumab was planned. A pretherapeutic *DPYD*-PGx was requested, and the patient was identified as DPYD*2A heterozygous. In addition, he was wild type for *MTHFR-C677T* and *MTHFR-A1298C*, heterozygous *TYMS TSER-2R/3R* and homozygous (GG) for *GSTP1-A313G*. The plasmatic UH2/U ratio was 3.22. Based on these results, a 50% dose reduction of 5-FU was planned for the first cycle of FOLFOX administration, according to the CPIC and DPWG guidelines. After the first cycle of treatment, the plasmatic 5-FU clearance was 474 ng/mL. Following three cycles of therapy, a stable disease was found, according to the RECIST criteria v1.1, and no adverse events were reported. The patient was still treated with the same doses of chemotherapeutic agents for an additional seven cycles of therapy. Grade 1 paraesthesia and mucositis and grade 2 HFS but no severe ADR were reported, and the CT scan demonstrated a stable disease. Afterward, the patient was treated up to the twelfth cycle with a FOLFOX regimen plus bevacizumab, still obtaining, at revaluation, a stable disease. Then, he was a candidate for a maintenance therapy with capecitabine plus bevacizumab. Following 16 cycles of this therapy, the patient reported grade 1 paraesthesia and mucositis, and no severe ADR were recorded.

Case 3 was a Caucasian 60-year-old male former smoker with no comorbidities. He had stage IV rectal adenocarcinoma with liver metastases. The tumour mutational profile did not identify mutations in either *KRAS*, *NRAS* or *BRAF*. Based on the tumour profile and stage, the patient was a candidate for a FOLFOX regimen plus cetuximab. A pretherapeutic *DPYD*-PGx was performed, and the patient was found heterozygous for *DPYD c2846A>T* SNP. Therefore, according to the CPIC and DPWG guidelines, he started chemotherapy with a 50% dose reduction of 5-FU. Moreover, he was homozygous (TT) for *MTHFR-C677T*, heterozygous *TYMS TSER-2R/3R* and heterozygous for *GSTP1-A313G*. The plasmatic UH2/U ratio was 1.77.

A grade 2 diffuse maculopapular rash was reported, and, based on such an ADR, the dose of cetuximab was also reduced to 50% for the second cycle of therapy. The plasmatic 5-FU clearance was 811 ng/mL—still high, notwithstanding the 5-FU dose reduction. The patient reported no improvement of the skin rash and grade 2 diarrhoea. At the third cycle of therapy with the same drugs doses, the 5-FU plasma level was 1093 ng/mL. Grade 1 nausea and grade 3 diarrhoea were reported. Based on these results, the 5-FU dose was further reduced by an additional 10% at the fourth cycle of therapy. However, the 5-FU plasma concentration was still high (1048 ng/mL), and grade 4 diarrhoea was reported. Hence, it was decided not to administer 5-FU in a continuous infusion, leaving the administration of 5-FU in bolus. Nevertheless, the 5-FU plasma concentration was still high (i.e., 934 ng/mL), and grade 3 diarrhoea was reported.

A CT scan showed a partial response according to the RECIST criteria with a reduction of hepatic lesions. It was decided to carry out a further cycle with oxaliplatin plus cetuximab.

After this cycle, the hepatic lesions were resected. After one month from surgery, a CT scan demonstrated the development of a new hepatic lesion. The patient was a candidate to start a new treatment with 5-FU plus irinotecan as a modified 5-FU, leucovorin and irinotecan (FOLFIRI) regimen, since 5-FU was administered with a 50% dose reduction and without continuous infusion. The patient performed six cycles of FOLFIRI plus bevacizumab. Grade 3 diarrhoea was reported. As a consequence, the 5-FU administration was stopped, and only irinotecan and bevacizumab were further administered. After four cycles of this treatment, the CT scan demonstrated a progression of the disease. The patient died after 11.2 months from starting treatment with irinotecan plus bevacizumab.

Table 1 and Table 2 report the main characteristics and the occurrence of grade ≥ 3 ADR of 3/11 and 8/11 clinical cases, respectively. Table 1 describes three clinical cases for whom either pretherapeutic *DPYD*-PGx or post-therapeutic *DPYD*-PGx were performed. As phenotypic characteristics, the UH2/U ratio values and plasmatic 5-FU clearance were reported.

A pretherapeutic *DPYD*-PGx was performed in two out of three cases, while one patient (case 1) had already started chemotherapy before requesting *DPYD*-PGx. Importantly, the patients were monitored during all treatment cycles.

Besides these three clinical cases, the history of other eight patients is briefly reported below, and their main characteristics are listed in Table 2.

All subjects were monitored for at least four treatment cycles. In two out of eight subjects, the *DPYD*-PGx was required after the occurrence of severe toxicity (post-therapeutic *DPYD*-PGx), while in six out of eight, pharmacogenetic testing was performed before the treatment started (pretherapeutic *DPYD*-PGx). All patients were identified as carriers of *DPYD* variants—precisely, four out of eight were DPYD*2A heterozygous, and four out of eight were *DPYD c.2846* heterozygous.

The two patients for whom the *DPYD*-PGx was performed after 5-FU administration experienced grade 3 ADR with a different timing, and both were then revealed as *DPYD*-variant carriers. More in detail, one patient with stage III gastric cancer, treated with FOLFOX, suffered from grade 3 vomit after the second cycle; he was then identified as DPYD*2A heterozygous and continued to be treated only with oxaliplatin. Moreover, the patient was homozygous (TT) for *MTHFR*-C677T, homozygous *TYMS TSER*-3R/3R and homozygous (AA) for *GSTP1*-A313G.

The other one with stage IV colon cancer, treated with FOLFIRI plus bevacizumab, showed grade 3 vomit after the eighth cycle of chemotherapy. The patient was identified as *DPYD c.2846* heterozygous, and the 5-FU dosage was halved. With regards to the other SNPs, the patient was homozygous (TT) for *MTHFR*-C677T, heterozygous *TYMS TSER*-2R/3R and wild type for UGT1A1*28 SNP. The latter polymorphism is routinely analysed in patients treated with irinotecan.

Conversely, in the other patients, a pretherapeutic *DPYD*-PGx was performed; thus they were treated with a starting halved dose of 5-FU, and no severe ADR were reported.

### 3.2. Systematic Review

A systematic review was performed to analyse the studies investigating the variability of responses to FP-based chemotherapy by *DPYD* genotyping combined with phenotyping methods and/or clinical monitoring.

Of the potential 112 articles assessed for eligibility, after considering the inclusion and exclusion criteria, 22 studies were included in the analysis [20,21,22,23,24,25,26,27,28,29,30,31,32,33,34,35,36,37,38,39,40,41]. Table 3 shows such studies subdivided with respect to the analysed *DPYD* polymorphisms (*DPYD*-PGx), the used phenotyping methods and the presence of clinical monitoring. A *DPYD-PGx*/clinical monitoring combination was present in 11, and *DPYD-PGx*/phenotyping in three, surveys. A *DPYD-PGx*/phenotyping/clinical monitoring combined approach was made in eight studies (Table 3). 

Among the studies with a DPYD-PGx/clinical monitoring combination, five out of eleven studies confirmed the importance of DPYD variants in predicting FP-related toxicity, although a too-short clinical monitoring was performed (only two treatment cycles) [21,23,29,31,39]. Two studies [24,34] analysed only DPYD*2A of the DPYD SNPs currently recommended. The first confirmed a strong association between DPYD*2A and a severe and potentially fatal toxicity [24]. The second did not analyse this type of association because of the too-low DPYD*2A allelic frequency found in the study population [34].

Lee et al., by testing 2886 patients, reported a significant association between DPYD*2A and DPYD c.2846A>T and grade ≥3 FP ADR [26]. Similarly, Cremolini et al., in a large cohort of colon cancer patients who were treated with FOLFIRI or FOLFOXIRI plus bevacizumab, demonstrated that DPYD*2A and DPYD c.2846A>T predicted FP-associated clinically relevant ADR [36]. Iachetta et al. analysed 668 out of 1827 patients enrolled in their study. The authors, first, confirmed the clinical relevance of DPYD c.2846A>T and DPYD*13 in predicting FP safety. Second, they found a significant association between DPYD*6 and severe neutropenia. Notably, no patients carrying DPYD*2A (1.7%) had started a FP-based treatment [38]. Negarandeh et al. screened the presence of DPYD c.2846A>T, DPYD*6 and DPYD*2A in a population of 73 Iranian patients. DPYD c.2846A>T and DPYD*6 were not found in the patient population analysed. However, a high allelic frequency for DPYD*2A (5.5%) was reported. Surprisingly, Negarandeh et al. did not find any significant association between DPYD*2A and severe toxicity [40]. 

A DPYD-PGx/phenotyping combined approach was performed in three studies that underlined the importance of complementing the DPYD-PGx with a phenotyping analysis. Gentile et al., by measuring the 5-FUDR (degradation rate) in peripheral blood mononuclear cells (PBMCs) utilising HPLC with MS/MS, found a significant correlation between several polymorphisms, including DPYD*2A and DPYD c.2846A>T, and this phenotyping marker [27]. Jacobs et al. determined 5-FU clearance in the plasma of patients treated with capecitabine, finding that the presence of DPYD*2A led to a 21.5% reduction in 5-FU elimination. Pallet et al. evaluated an approach based on the combination of the plasmatic UH2/U ratio and uracil concentration with genotyping of the four recommended DPYD SNPs. The main finding of this study was that complementing the DPYD-PGx with the plasmatic UH2/U ratio increased the possibility to identify patients at higher risks of severe FP-related toxicity [41]. Among the studies that performed a DPYD-PGx/phenotyping approach with clinical monitoring, four out of eight studies reported information about FP-related ADR until a maximum of three treatment cycles. 

Joerger et al. confirmed the importance of DPYD genotyping to identify patients at high risks of severe FP-related toxicity. Unfortunately, because of the low sample size of the study, they did not show conclusive results about the usefulness of plasmatic 5-FU clearance determination in improving the predictive potential of DPYD-PGx [28]. 

Galarza et al. found that salivary and plasmatic UH2 concentrations were inversely correlated with the ADR grade. However, given the low number of patients enrolled in the study, no DPYD variant allele carriers were identified [30].

Boisdron et al. conducted a phase II study in 85 patients to test the efficacy of a pharmacogenetic-guiding dosing approach combined with the UH2/U ratio measurement. Despite a very large increase in drug dosages, a low incidence of severe ADR was shown in patients who used a guiding dosing approach. However, also in this case, it was not possible to conclude if this phenotyping analysis enhanced the predictability of DPYD genotyping because of the low sample size of the study [32]. Etienne et al. failed to demonstrate a correlation between DPYD variants and the plasmatic UH2/U ratio values. The authors concluded that only an extension of the genetic panel may improve the performance of DPYD-PGx for predicting severe and life-threatening ADR associated with capecitabine [33]. 

Kuilenburg et al. measured the UH2/U ratio in PBMCs as an indirect assessment of DPYD activity. They demonstrated that patients with a low DPD activity experienced a more rapid onset of toxicity as compared to those with a normal enzymatic activity. Moreover, grade 4 neutropenia occurred in a substantial percentage (55%) of the patients with a decreased DPD activity as compared to that (13%) of subjects with a normal DPD activity. Notably, eleven out of fourteen patients suffering from severe ADR with a decreased enzymatic activity were identified as carriers of DPYD polymorphisms. In particular, six, four and one out of eleven patients carried DPYD*2A, DPYD*9A and DPYD*6 in homozygosis, respectively [20]. Kristensen et al. also showed a significant correlation between the plasmatic UH2/U ratio and the presence of DPYD*2A [22].

Finally, in a prospective study, Henricks et al. analysed all the four recommended DPYD SNPs and performed two phenotyping tests by measuring the UH2/U ratio in PBMCs and plasmatic 5-FU pharmacokinetics (PK) by UHPLC-MS/MS. The patients carrying DPYD c.1236G>A and DPYD c.2846A>T were more likely to manifest FP-related severe toxicity as compared to wild-type subjects. In addition, the mean DPD enzyme activity was significantly lower in patients bearing these two genetic variants, as well as DPYD*2A, as compared to other patients. Only one patient carrying DPYD*13 showed a 60% DPD activity reduction. This patient was treated with a reduced 5-FU dosage for three treatment cycles, and no severe ADR occurred. Based on these results, the authors confirmed that a dose reduction of 50% in DPYD*2A and DPYD*13 carriers is appropriate, as issued in the PGx guidelines [35].

## 4. Discussion

Fluoropyrimidines (FP) are the most-prescribed antineoplastic drugs in the world and represent the mainstay therapy for colorectal cancer. Unfortunately, 10–30% of the treated patients suffer from severe FP-related ADR [1].

The association between four *DPYD* variants and FP toxicity is now widely recognised, and it is also largely accepted that the pretherapeutic guiding dosing model based on the *DPYD*-PGx is a valid and cost-effective approach to manage FP-based chemotherapy. As a matter of fact, FP are included among the drugs with a pharmacogenetic warning [5], and the CPIC and DPWG guidelines are now followed in several countries, including Italy.

However, *DPYD*-PGx is not yet routinely performed, and one of the aims of these consortia is to overcome the barriers preventing the implementation of the PGx into clinical practice. In March 2019, the Pharmacovigilance Risk Assessment Committee (PRAC) of the European Medicines Agency (EMA) recommended to perform *DPYD*-PGx before starting treatment with FP. As in the CPIC and DPWG guidelines, the PRAC stated that 5-FU, capecitabine or tegafur must be avoided in the presence of a complete DPD deficiency. In the case of a partial DPD deficit, a reduced starting dose of FP should be considered [42].The PRAC recommendation was transferred to the Committee for Medicinal Products for Human Use (CHMP), and, on 7 July 2020, the EMA Commission raised a final decision that the *DPYD*-PGx should be performed in naïve oncological patients prior to starting a treatment with FP [43].

A substantial percentage of patients with a *DPYD* variant associated with a deficit of DPD activity show grade > 2 toxicity [9]. For instance, it has been estimated that 73% of the patients who are carriers of DPYD*2A suffer from grade ≥ 3 toxicity [24]. Therefore, the upfront *DPYD*-PGx has a crucial impact on the management of chemotherapy given that it may prevent 20–30% of life-threatening or lethal FP-related toxicity in Caucasian patients, revealing also a cost-effective approach [24,44].

In this regard, case 1 suffered from grade 3 neutropenia and grade 3 mucositis before the *DPYD*-PGx was required, which then revealed the presence of DPYD*2A SNP. Notably, once the PGx results were obtained, the patient received 50% of the 5-FU dosage, and at the fourth and following cycles, there was no evidence for severe ADR.

The importance of performing a pretherapeutic *DPYD*-PGx is confirmed by the history of two other patients who had experienced grade 3 toxicity, with a different timing, before the test was requested. One of them was identified as DPYD*2A heterozygous and the other one as *DPYD c.2846* heterozygous. It is possible to conclude that FP-related severe toxicity may occur anytime during the therapy. The DPYD*2A, which is a no-function variant, could be associated with a more premature ADR when compared with *DPYD c.2846A>T* SNP, which is classified as reduced-function variant. In fact, the subject bearing the DPYD*2A suffered from grade 3 vomit at the second cycle, while the other patient, who was the carrier of the *DPYD c.2846*, suffered from the same toxicity but at the eighth cycle of chemotherapy.

Notably, it has been estimated that a 30–50% of the patients treated with FP have no DPD deficit attributable to the four recommended *DPYD* SNPs, yet suffer from severe ADR [45]. In fact, several factors concur to determine the variability of responses to FP-based treatments. Among them, other polymorphisms in *DPYD*, and in other genes encoding enzymes involved in the pathway of the FP and, also, FP-associated drugs, may play an important role. In this regard, several studies have shown an association between the *TYM-TSER* 28-bp variant (in the gene encoding the FP molecular target) and FP-related toxicity [12,46]. Moreover, an increased risk of severe toxicity has been shown in patients carrying rs183205964 in the promoter enhancer region of *TYMS* [46].

Finding the relationship between FP and specific ADR can be very difficult also, because FP are often administered with both traditional and biologic antineoplastic agents, such as cetuximab and bevacizumab. These drugs, as well as oxaliplatin and irinotecan, can cause diarrhoea and myelosuppression [47,48,49,50] similar to FP, and, as already mentioned, there are several genetic variants that are associated to these ADR and others.

The MTHFR is a critical enzyme involved in the synthesis of purine and thymidine and, in general, folate homeostasis. Two common SNPs (i.e., *MTHFR-C677T* and -*A1298C*), associated with a decrease of the MTHFR enzymatic activity, have been proposed as predictive factors of the response to cytotoxic agents, including FP, raltitrexed and methotrexate [51]. In addition, these two SNPs may exert a synergistic effect on the MTHFR activity [11]. A recent metanalysis failed to recognise the *MTHFR* polymorphisms (neither *MTHFR-C677T* nor -*A1298C*) as predicting factors for the response to FP-based treatment [52]. The data often contrast each other because of several reasons, including the type of cancer, dietary folate levels, therapy regimen and saturation level of the enzyme [51].

Oxaliplatin is mainly metabolised by glutathione-S-transferase P1 (GSTP1), and the SNP in this gene (i.e., *GSTP1 A313G*, *Ile105val* and rs1695) has been associated with severe ADR. Most of the evidence regards the occurrence of cumulative neuropathy associated with such a polymorphism [12,53], but the presence of at least one variant allele has been associated also with grade 3 or 4 gastrointestinal and haematological toxicity [54].

In a recent metanalysis, Lv et al. found an association between the *GSTP1* rs1695 SNP and granulocytopenia induced by platinum derivatives [55]. Another metanalysis failed to demonstrate that this polymorphism can be considered a reliable predictive factor of oxaliplatin-related severe neurotoxicity [56]. At present, the data are inconclusive; therefore, only large, well-designed clinical trials will be able to clarify this issue.

The *TYMS-TSER* 28-bp tandem repeat is one of the more promising candidates as a genetic factor responsible for FP-related ADR. However, as reported in the last CPIC update, its clinical relevance is still debated [5].

Lecomte et al. found that patients who were carriers of the double-repeat allele (i.e., *TYMS-TSER-2R*) had more severe FP-associated ADR than the homozygous *-3R/3R*. In particular, the authors reported that patients bearing the *-2R/2R* genotype were 20 times more likely to suffer from severe toxicity compared with those who are carriers of the *-3R/3R* genotype. This could be related to a decreased TYMS mRNA expression in the normal tissue of subjects bearing a 2R/2R genotype that may lead to increased thymidylate synthase (TS) inhibition by FP treatment [11]. These findings refer to the somatic variant, while the data regarding the same germline polymorphism, which is identifiable by means of a peripheral blood sample, are inconclusive. Obviously, if the germline variant could be considered a predictive parameter like the somatic ones, a less-invasive method would be available to individualise the FP-based treatment in naïve patients. Several recent studies have suggested that the *TYMS TSER* germline polymorphism may be useful to prevent toxicity [57], such as hand-foot syndrome and other ADR types, including haematological and gastrointestinal ones [58]. With regards to the clinical cases described in Table 1, case 1 was homozygous *TYMS TSER-2R/2R* and case 2 and 3 were heterozygous *2R/3R*.

Undoubtedly, a larger panel of genetic variants is needed. On the other hand, further studies should be performed to support the regulatory authorities to decide whether and which new polymorphisms should be added to the four recommended *DPYD* SNPs in the PGx guidelines. Researchers’ efforts are addressed to find the best phenotyping methods to complement the *DPYD*-PGx. Among the different approaches proposed until now, the analysis of the 5-FU pharmacokinetic parameters was evaluated even before the *DPYD*-PGx introduction. In the eighties, in fact, a high individual variability of the plasmatic 5-FU clearance was demonstrated, and the existence of a correlation between the 5-FU levels and FP-related toxicity had already been highlighted [59].

Nowadays, it is possible to determine the 5-FU clearance using sophisticated techniques, such as HPLC coupled with tandem mass spectrometry (LC-MS/MS), which provides the precious opportunity to carry out TDM to adjust the therapy cycle-by-cycle where appropriate. Nonetheless, this approach is not always feasible in hospital settings and is unable to detect a DPD deficit prior to starting the treatment, thus preventing an early severe toxicity. Moreover, a 5-FU pharmacokinetic analysis aims at measuring the area under the curve (AUC) of the 5-FU plasma levels at the steady state (i.e., 2 h after the drug administration) and requires collecting multiple blood samples [14].

The CPIC and DPWG guidelines recommend to perform the *DPYD*-PGx and to treat the heterozygous patients with a dose reduction at the first cycle of chemotherapy and then titrate the dosage while performing a careful clinical monitoring and possibly TDM [5,6].

An ideal management workflow should consent to optimise FP-based therapy before treatment using the least invasive method; therefore, the researchers’ efforts have been addressed in this direction.

The measure of the plasmatic UH2/U ratio has been proposed as a suitable approach, but its validity is brought up for discussion. Indeed, some studies have demonstrated that this parameter well-correlates with 5-FU clearance and FP-related toxicity [22], while others have suggested that it can be considered a valid predictive factor during 5-FU administration alone [25]. Notably, determining the UH2/U ratio in peripheral blood mononuclear cells (PBMCs) seems to be the best method, but, unfortunately, it is a time-consuming procedure and requires radiolabelled reagents and large volumes of blood [20,35,60].

Importantly, it seems that only a low plasmatic UH2/U ratio is a good predictive factor. For instance, Gamelin et al. suggested that a value less than 1.8 really helps to identify the patients at higher risk of FP-related toxicity [61]. To this line, Kristensen et al. studied 24 patients treated with FP who experienced FP severe-related toxicity, finding that 21 out of 24 subjects had a UH2/U ratio ≤4. They stated that the average UH2/U ratio decreased with the toxicity grade and proposed the value of 4 as the cut-off value [22]. Other authors have fixed the cut-off value at 6, specifying that the patients carrying one functional and one nonfunctional allele of *DPYD* (i.e., heterozygous) have a UH2/U ratio ranging from 1.5 to 6, while those who are carriers of two nonfunctional alleles (i.e., homozygous) have a value close to 0, corresponding to a complete DPD deficiency [62].

Besides the patients described in Table 1, we have measured the plasmatic UH2/U ratio in another eight subjects who are all heterozygous for DPYD*2A or *DPYD c.2846A>T*. We did not perform the pharmacokinetic analysis on these patients for several reasons (e.g., unavailability of the patients consents and changes of the therapy). It is important to underline that in six out of eight patients for whom a pretherapeutic *DPYD*-PGx was performed and the starting 5-FU dosage was halved, no severe toxicity was recorded (Table 2).

In the 11 patients we examined, the UH2/U ratio ranged between 1.77 and 7.38.

The fluctuation of the UH2/U ratio values as a predictive factor could reflect the variability of the DPD deficit that may range from 30% to 70% in the heterozygous patients. Nonetheless, the plasmatic UH2/U ratio could contribute to identifying, before beginning treatment, the subjects who are at-risk of severe or life-threatening ADR. Case 3 (reported in Table 1) exemplifies the complexity of the management of FP-based therapy. In fact, he had a very low (i.e., 1.7) plasmatic UH2/U ratio and continued suffering from severe toxicity despite the reduction of the 5-FU dosage. It is mandatory to bear in mind that this ratio remains a surrogate marker of DPD activity, and even if it was a more sensitive and specific parameter, several other variables may influence both the efficacy and tolerability of FP-based chemotherapy.

This is the reason why a combined genotyping/phenotyping approach, together with careful clinical monitoring, is the best way to personalise and optimise the therapy. Several studies have used this kind of approach, suggesting that phenotyping analyses, especially those based on the plasmatic 5-FU clearance and/or UH2/U ratio measurement, could successfully complement the *DPYD*-PGx in predicting FP-related toxicity. This is clearly shown by our systematic literature search (Figure 1 and Table 3).

As shown in Table 3, eight studies have combined *DPYD*-PGx with phenotyping methods and clinical monitoring, but only two out of eight have provided satisfactory clinical monitoring [28,35]. Indeed, to perform daily careful, clinical monitoring during all patients’ treatment cycles is very arduous. In fact, among these studies, three out of eight reported ADR only for two cycles [22,25,33], one out of eight monitored the toxicity until the third cycle [30] and two out of eight made clinical monitoring for two-to-three months [20,32].

Moreover, as shown in Table 3, three studies have performed a combined *DPYD*-PGx/phenotyping [27,37,41] approach, and eleven carried out the *DPYD*-PGx together with clinical monitoring. However, among these, only 2/11 genotyped all the four *DPYD* SNPs recommended in the CPIC and DPWG guidelines [29,39]; the other studies performed at least one of such SNPs, together with other *DPYD* genetic variants [21,22,23,24,25,26,27,28,29,30,31,32,33,34,35,36,37,38,39,40].

The main limitations of the studies performed until now are the lack of clinical monitoring throughout the entire course of chemotherapy and the scarce sample size. Moreover, the missing of the screening of one or more of the four *DPYD*-SNPs now recommended and the heterogeneity in the choice of other polymorphisms potentially useful to implement genotyping do not allow reaching conclusive results.

## 5. Conclusions

The pretherapeutic DPYD-PGx represents an essential approach to personalise FP-based chemotherapy, minimising the risk of severe and life-threatening toxicity. This is confirmed by both the clinical cases here described and the literature data.

Nowadays, the regulatory agencies recommend carrying out the *DPYD*-PGx, including four DPYD polymorphisms (i.e., rs3918290, rs55886062, rs67376798 and rs75017182, HapB3) in patients who need to be treated with FP.

Despite that these DPYD variants are strongly associated with treatment toxicity, other genetic and nongenetic factors concur to determine the variable response to FP-based chemotherapy.

Initiating the FP with a reduced dosage is not a suitable option, because DPD deficits may range from 30% to 70% in the heterozygous patients who would experience a dangerous period of undertreatment.

A pretherapeutic DPYD-PGx offers the possibility to avoid early ADR. Nonetheless, severe and even fatal FP-related toxicity may happen anytime during the therapy also in subjects having no DPD deficit attributable to the four recommended DPYD SNPs.

On the other hand, measuring the plasmatic 5-FU clearance—currently, the best method to perform TDM—does not permit to diagnose a possible DPD deficit prior to starting the treatment.

**Figure 2:**
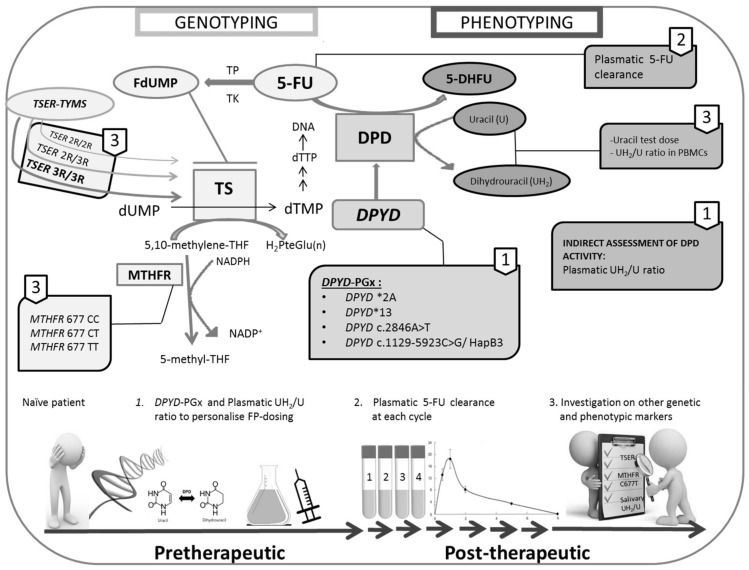
This figure illustrates the main enzymatic reactions involved in the fluoropyrimidines (FP) pathway. The importance to perform, together with careful clinical monitoring, a genotyping/phenotyping combined approach is shown by using panels and arrows. The four recommended *DPYD*-SNPs, including in the *DPYD*-PGx, are listed, together with the other polymorphisms proposed as potential predictive factors of FP-related toxicity. Phenotyping methods (e.g., UH2/U ratio measured in plasma or PBMCs), which potential in predicting FP safety are currently under evaluation, are also described. An ideal flowchart to manage patients eligible for FP-based therapy is shown at the bottom of the figure. *DPYD*-PGx and the plasmatic UH2/U ratio have to perform one time (continuous line), while the plasmatic 5-FU clearance is throughout the entire course of the therapy (dotted line). The numbers located on the panels are the same reported in the flowchart in order to underline which genotyping and phenotyping markers may be used together to optimise the diagnosis and management of patients. Abbreviations: DPD, dihydropyrimidine dehydrogenase; *DPYD*-PGx, *DPYD*-pharmacogenomics; 5-FU, fluorouracil; DHFU, 5-fluoro-dihydrouracil; TP, thymidine phosphorylase; TK, thymidine kinase; FdUMP, 5-fluorodeoxyuridine monophosphate; TS, thymidylate synthase; dUMP, deoxyuridine monophosphate; dTMP, deoxythymidine monophosphate; dTTP, deoxythymidine triphosphate; 5,10-methylene-THF, 5,10-methylene-tetrahydrofolate; H2PteGlu(n), dihydrofolic acid; MTHFR, methylene-tetrahydrofolate reductase; 5-methyl-THF, 5,10-methyl-tetrahydrofolate; NADPH, nicotinamide adenine dinucleotide phosphate and *TYMS-TSER*, thymidylate synthase-thymidylate synthase enhancer region.

Therefore, because both genetic and phenotypic tests show advantages and disadvantages, a combined genotyping/phenotyping approach, together with careful and continuous clinical monitoring, is the best diagnostic method to optimise the therapy with FP (Figure 2).

An accurate genotypic/phenotypic characterisation of each patient is essential not only to prevent severe toxicity associated with the cytotoxic agents but, also, to determine patients’ benefit-risk profiles to begin early the best therapeutic approach. This is of particular interest considering the current possibility to treat certain patients with anticancer agents, such as immune checkpoint inhibitors, that are changing the cancer therapeutic paradigm.

## Figures and Tables

**Table 1:** Reports of the main characteristics of three patients with the occurrence of grade ≥ 3 ADR.

Pt	Sex	Age (years)	Tumor Type and Stage	Chemotherapy Regimen	Pre-Therapeutic *DPYD*-PGx	Post-Therapeutic *DPYD*-PGx	*DPYD* Genotype	UH2/U Ratio	5-FU Dosage	5-FU Clearance	ADR ≥ 3	Total Toxicity
1	M	55	CRC (metastatic)	Folfox plus cetuximab	/	Yes (C4)	Heterozygous for DPYD*2A	4.52	C1-C3: 100% C4-C6: 50%	950 ng/mL (C4) 400 ng/mL (C6)	G3 mucositis (C3) G3 neutropenia (C3) (neuthrophils: 830.58 /mm^3^)	7
2	M	48	CRC (metastatic)	Folfox plus bevacizumab	Yes	/	Heterozygous for DPYD*2A	3.22	C1-C8: 50%	474 ng/mL (C1)	/	6
3	M	60	Rectal cancer (metastatic)	Folfox plus cetuximab)	Yes	/	Heterozygous for c.2846A>T	1.77	C1-C3: 50% C4: 40% C5-C6: 40% (only bolus)	811 ng/mL (C2) 1093 ng/mL (C3) 1048 ng/mL (C4) 934 ng/mL (C5)	G3 diarrhoea (C3) G4 diarrhoea (C4) G3 diarrhoea (C5)	3

Pt, patient; M, male; CRC, colorectal cancer; FOLFOX, 5-fluorouracil plus leucovorin plus oxaliplatin; *DPYD*-PGx, *DPYD* pharmacogenetics; 5-FU, 5-fluorouracil; C, cycle; plasmatic UH2/U ratio, dihydrouracil/uracil ratio; ADR, adverse drug reaction; G, grade and total toxicity, number of ADR regardless of the grade of severity.

**Table 2:** Reports of 8 clinical cases for whom either pretherapeutic *DPYD*-PGx or post-therapeutic *DPYD*-PGx were performed. As phenotypic characteristics, the UH2/U ratio values were reported.

Pt	Sex	Age (Years)	Tumor Type and Stage	Chemotherapy Regimen	Pre-Therapeutic *DPYD*-PGx	Post-Therapeutic *DPYD*-PGx	*DPYD* Genotype	UH2/U Ratio	5-FU Dosage	ADR ≥3
1	F	63	Stomach cancer (locally advanced)	Folfox	/	Yes (C2)	Heterozygous for DPYD*2A	7.09	C1-C2: 100% C3: 5-FU withdrawal	G3 vomit (C2)
2	M	43	CRC (metastatic)	Folfiri with bevacizumab	/	Yes (C8)	Heterozygous for c.2846A>T	3.88	C1-C8: 100% C9: 50%	G3 vomit (C8)
3	M	63	Kidney cancer (metastatic)	Xeloda	yes	/	Heterozygous for c.2846A>T	6.57	C1-C6: 50%	/
4	M	68	CRC (metastatic)	Xelox	yes	/	Heterozygous for c.2846A>T	4.4	C1-C7: 50%	/
5	M	78	CRC (local)	Xelox	yes	/	Heterozygous for c.2846A>T	3.37	C1-C2: 50%	/
6	M	72	CRC (locally advanced)	Xelox	yes	/	Heterozygous for DPYD*2A	5.15	C1-C8: 50%	/
7	F	52	Vulva carcinoma (local)	Xeloda with cisplatin	yes	/	Heterozygous for DPYD*2A	7.38	C1-C5: 50%	/
8	M	76	Rectosigmoid cancer (locally advanced)	Folfox	yes	/	Heterozygous for DPYD*2A	2.44	C1-C5: 50%	/

Pt, patient; M, male; F, female; CRC, ColoRectal Cancer; FOLFOX, 5-Fluorouracil plus leucovorin plus oxaliplatin; FOLFIRI, 5-Fluorouracil plus leucovorin plus irinotecan; XELOX, capecitabine plus oxaliplatin; XELODA, Capecitabine; DPYD-PGx, DPYD pharmacogenetics; UH2/U RATIO, dihydrouracil/uracil ratio; ADR, Adverse Drug Reaction; C, cycle; G, Grade.

**Table 3:** The table reports the studies included in the systematic review and subdivided into three groups: DPYD-PGx/clinical monitoring combination, DPYD-PGx/phenotyping and DPYD-PGx/phenotyping/clinical monitoring. Abbreviations: PBMC, peripheral blood mononuclear cells; HPLC-UV, high-performance liquid chromatography-UV detector; LC-MS/MS, liquid chromatography-tandem mass spectrometry; 5-FUDR, 5-FU degradation rate; UHPLC-MS/MS, ultra-high-performance liquid chromatography-tandem mass spectrometry; PK, pharmacokinetics; FP, fluoropyrimidines; DPD, dihydropyrimidine dehydrogenase; UH2/U ratio, dihydrouracil/uracil ratio and AAS, atomic absorption spectrometry.

First Author‘s Name (Published Year)	Enrolled Patients (n)	Outcomes	*DPYD*-PGx/Clinical Monitoring	*DPYD*-PGx/Phenotyping	*DPYD*-PGx/Phenotyping/Clinical Monitoring
Kuilenburg et al. (2000) [20]	37	*DPD* activity and overall toxicity; *DPYD* genotyping in patients with reduced DPD activity.			DPYD*2A, c.2846A>T, DPYD*6, DPYD*9A, c.496A>G/ UH2/U ratio in PBMC/ADR until two treatment months.
Schwab et al. (2008) [21]	683	Overall toxicity; *DPYD, TYMS, MTHFR* genotyping; sequencing of *DPYD* exome; influence of sex and promoter methylation on DPD expression in human liver.	DPYD*2A, c.2846A>T, c.623G>T, DPYD*4, DPYD*6, and c.2858G>C/ ADR reported until the second cycle of treatment.		
Kristensen et al.(2010) [22]	68	Relationship between UH_2_/U plasma ratio and 5-FU-related early toxicity; relationship between 5-FU concentration and toxicity; IVS14+1G>A mutation screening.			DPYD*2A/ UH_2_/U ratio in plasma 5-FU clearance by HPLC-UV/ ADR reported until the second cycle of treatment.
Deenen et al. (2011) [23]	568	Relationships between SNPs and toxicity, SNPs and dose modification of capecitabine, *DPYD* haplotypes and toxicity, *DPYD* SNPs and haplotypes and survival.	DPYD*2A, c.2846A>T and c.1236G>A [*HapB3*]/ ADR reported until the second cycle of treatment.		
Deenen et al. (2016) [24]	2038	Feasibility, safety and cost of DPYD*2A genotype-guided dosing.	DPYD*2A/ADR reported until the sixth cycle of treatment.		
Sistonen et al. (2014) [25]	28	Relationship between UH_2_/U plasma ratio and *DPYD* genetic variation; plasma concentration of 5-FU and corresponding AUC; toxicity.			c.234-123G>C, c.496A>G, c.775A>G, c.1129-5923C>G [*Hap B3*], DPYD*13, DPYD*2A and c.2846A>T/ UH_2_/U ratio in plasma- 5-FU clearance by LC-MS/MS/ ADR reported until the second cycle of treatment.
Lee et al.(2014) [26]	2886	Relationship between *DPYD* variants and toxicity.	DPYD*2A, DPYD*13, c.2846A>T/ ADR until the twelfth cycle of treatment.		
Gentile et al. (2015) [27]	156	Correlation between degradation rate of 5-FU with detected SNPs.		DPYD*2A, DPYD*13, c.2846A>T/5-FUDR assay in PBMC by HPLC-MS/MS	
Joerger et al. (2015) [28]	140	Quantitative effect of 44 gene polymorphism in 16 drug pathway associated genes on progression free survival (PFS), on chemotherapy toxicity, on objective response rate (ORR), on overall survival (OS).			DPYD*13, DPYD*2A, c.2846A>T, DPYD*9A, c.1896T>C/5-FU clearance by AAS and HPLC/ADR until disease progression.
Lunenburg et al. (2016) [29]	275	Evaluation of requests of prospective *DPYD* screening and results with a dose recommendation; estimation of the follow up of the dose recommendations.	DPYD*2A, DPYD*13, c.2846A>T, c.1236G>A [HapB3]/ ADR reported until the second cycle of treatment.		
Galarza et al. (2016) [30]	60	Estimation of the use of plasma and saliva; Uracil to UH_2_ metabolic ratio and *DPYD* genotyping.			*DPYD* *2A, *13, c.557A>G, *DPYD* *7/ UH_2_/U ratio in plasma/ ADR reported until the third cycle of treatment.
Milano et al. (2016) [31]	243	Sequencing of *DPYD* exome and frequence of G3, G4 toxicity over cycle 1-2.	DPYD*2A, DPYD*13, c.2846A>T, c.1774C>T, c.1475C>T, D342G/ ADR reported until the second cycle of treatment.		
Boisdron-Celle et al.(2017) [32]	85	*UGT1A1* and *DPYD* genotyping; UH_2_/U ratio; follow up of efficacy and tolerance.			DPYD*2A, DPYD*13, c.2846A>T, DPYD*7/ UH_2_/U ratio in plasma/ADR every two weeks until three months.
Etienne-Grimaldi et al.(2017) [33]	243	*DPYD* sequencing; relationship between toxicity and *DPYD* variants; *DPD* phenotyping.			DPYD*2A, DPYD*13, c.2846A>T/ UH_2_/U ratio in plasma/ ADR reported until the second cycle of treatment.
Liu et al.(2017) [34]	661	Relationship between *UGT1A1* and *DPYD* polymorphism and incidence of severe neutropenia and diarrhea; relationship between *UGT1A1* and *DPYD* variants and objective response rate, disease control rate, overall and progression free survival.	DPYD*5, c.1896 T > C, and DPYD*2A/ ADR reported every two-three cycles or whenever patient’s condition changed.		
Henricks et al.(2018) [35]	1181	Frequency of severe overall FP-related toxicity; pharmacokinetics of fluoropyrimidines in *DPYD* variant allele carriers; *DPD* enzyme activity; cost analysis on individualised dosing by upfront *DPYD* genotyping.			DPYD*2A, c.2846A>T, DPYD*13 and c.1236G>A [*Hap B3*]/ UH_2_/U ratio in PBMC/PK data by UHPLC-MS/MS/ADR until toxicity resolution.
Cremolini et al. (2018) [36]	443	Relationship between *DPYD* and *UGT1A1* genotyping and toxicity.	DPYD*2A, *13, c.2846A>T/ADR reported until the fourth cycle of treatment.		
Jacobs et al.(2019) [37]	237	Pharmacokinetics of capecitabine and 5-FU in *DPYD* variant allele carriers.		DPYD*2A, c.2846A>T, c.1236G>A [*HapB3*]/5-FU clearance by LC MS/MS.	
Iachetta et al.(2019) [38]	1827	Relationship between *DPYD* and toxicity.	DPYD*13, DPYD*2A, c.2846A>T, DPYD*6 /ADR reported until the eleventh cycle of treatment.		
Kleinjan et al. (2019) [39]	185	*DPYD* genotyping and toxicity.	DPYD*2A, c.2846A>T, DPYD*13 and c.1236G>A [HapB3] /ADR reported until the second cycle of treatment.		
Negarandeh et al.(2020) [40]	88	Relationship between *DPYD* genotyping and toxicity.	DPYD*2A, c.2846A>T, DPYD*6/ADR reported following 227 cycles for 88 patients.		
Nicolas Pallet et al.(2020) [41]	5886	Relationship between *DPYD* genotyping and [U] and UH_2_/U ratio in plasma.		DPYD*2A, DPYD*13, c.2846A>T, c.1236G>A [HapB3]/ [U] and UH_2_/U ratio in plasma.	

## Abbreviations

FP	Fluoropyrimidines
DPD	Dihydropyrimidine Dehydrogenase
*DPYD*-PGx	*DPYD*-Pharmacogenomics
PCR	Polymerase Chain Reaction
5-FU	5-Fluorouracil
ADR	Adverse Drug Reaction
HFS	Hand-Foot Syndrome
SNPs	Single-Nucleotide Polymorphisms
CPIC	The Clinical Pharmacogenetics Implementation Consortium
DPWG	Dutch Pharmacogenetics Working Group
DPYD-AS	*DPYD*-Activity Score
TDM	Therapeutic Drug Monitoring
MTHFR	Methylene-Tetrahydrofolate Reductase
TYMS-TSER	Thymidylate Synthase-Thymidylate Synthase Enhancer Region
GSTP1	Glutathione S-Trasferase-p1
UH2/U ratio	Dihydrouracil/Uracil Ratio
CTC-AE	Common Terminology Criteria for Adverse Events
RECIST	Response Evaluation Criteria in Solid Tumours
HPLC	High-Performance Liquid Chromatography
UHPLC-MS/MS	Ultra-High-Performance Liquid Chromatography-Tandem Mass Spectrometry
MeSH	Medical Subject Heading
PRISMA	Preferred Reporting Items for Systematic reviews and Meta-Analyses
FOLFOX	5-FU, Leucovorin and Oxaliplatin
CT scan	Computed Tomography Scan
FOLFIRI	5-FU, Leucovorin and Irinotecan
PRAC	Pharmacovigilance Risk Assessment Committee
EMA	European Medicines Agency
CHMP	Committee for Medicinal Products for Human Use
AUC	Area Under the Curve
LC-MS/MS	Liquid Chromatography-Tandem Mass Spectrometry
PBMCs	Peripheral Blood Mononuclear Cells
